# Relationships Between Tibiofemoral Contact Forces and Cartilage Morphology at 2 to 3 Years After Single-Bundle Hamstring Anterior Cruciate Ligament Reconstruction and in Healthy Knees

**DOI:** 10.1177/2325967117722506

**Published:** 2017-08-31

**Authors:** Young People With Old Knees Research Team, David John Saxby, Adam L. Bryant, Xinyang Wang, Luca Modenese, Pauline Gerus, Jason M. Konrath, Kim L. Bennell, Karine Fortin, Tim Wrigley, Flavia M. Cicuttini, Christopher J. Vertullo, Julian A. Feller, Tim Whitehead, Price Gallie, David G. Lloyd

**Affiliations:** *Investigation performed at School of Allied Health Sciences, Griffith University, Gold Coast, Australia; Centre for Health, Exercise and Sports Medicine, University of Melbourne, Melbourne, Australia; and the Department of Epidemiology and Preventive Medicine, Monash University, Melbourne, Australia*

**Keywords:** knee contact forces, anterior cruciate ligament reconstruction, meniscus, tibiofemoral cartilage, osteoarthritis

## Abstract

**Background:**

Prevention of knee osteoarthritis (OA) following anterior cruciate ligament (ACL) rupture and reconstruction is vital. Risk of postreconstruction knee OA is markedly increased by concurrent meniscal injury. It is unclear whether reconstruction results in normal relationships between tibiofemoral contact forces and cartilage morphology and whether meniscal injury modulates these relationships.

**Hypotheses:**

Since patients with isolated reconstructions (ie, without meniscal injury) are at lower risk for knee OA, we predicted that relationships between tibiofemoral contact forces and cartilage morphology would be similar to those of normal, healthy knees 2 to 3 years postreconstruction. In knees with meniscal injuries, these relationships would be similar to those reported in patients with knee OA, reflecting early degenerative changes.

**Study Design:**

Cross-sectional study; Level of evidence, 3.

**Methods:**

Three groups were examined: (1) 62 patients who received single-bundle hamstring reconstruction with an intact, uninjured meniscus (mean age, 29.8 ± 6.4 years; mean weight, 74.9 ± 13.3 kg); (2) 38 patients with similar reconstruction with additional meniscal injury (ie, tear, repair) or partial resection (mean age, 30.6 ± 6.6 years; mean weight, 83.3 ± 14.3 kg); and (3) 30 ligament-normal, healthy individuals (mean age, 28.3 ± 5.2 years; mean weight, 74.9 ± 14.9 kg) serving as controls. All patients underwent magnetic resonance imaging to measure the medial and lateral tibial articular cartilage morphology (volumes and thicknesses). An electromyography-driven neuromusculoskeletal model determined medial and lateral tibiofemoral contact forces during walking. General linear models were used to assess relationships between tibiofemoral contact forces and cartilage morphology.

**Results:**

In control knees, cartilage was thicker compared with that of isolated and meniscal-injured ACL-reconstructed knees, while greater contact forces were related to both greater tibial cartilage volumes (medial: *R* ^2^ = 0.43, β = 0.62, *P* = .000; lateral: *R* ^2^ = 0.19, β = 0.46, *P* = .03) and medial thicknesses (*R* ^2^ = 0.24, β = 0.48, *P* = .01). In the overall group of ACL-reconstructed knees, greater contact forces were related to greater lateral cartilage volumes (*R* ^2^ = 0.08, β = 0.28, *P* = .01). In ACL-reconstructed knees with lateral meniscal injury, greater lateral contact forces were related to greater lateral cartilage volumes (*R* ^2^ = 0.41, β = 0.64, *P* = .001) and thicknesses (*R* ^2^ = 0.20, β = 0.46, *P* = .04).

**Conclusion:**

At 2 to 3 years postsurgery, ACL-reconstructed knees had thinner cartilage compared with healthy knees, and there were no positive relationships between medial contact forces and cartilage morphology. In lateral meniscal-injured reconstructed knees, greater contact forces were related to greater lateral cartilage volumes and thicknesses, although it was unclear whether this was an adaptive response or associated with degeneration. Future clinical studies may seek to establish whether cartilage morphology can be modified through rehabilitation programs targeting contact forces directly in addition to the current rehabilitation foci of restoring passive and dynamic knee range of motion, knee strength, and functional performance.

Anterior cruciate ligament (ACL) rupture is a prevalent,^54^ debilitating,^27^ and costly^25^ intra-articular knee injury. Reconstruction of the ruptured ACL (ACLR) is cost-effective,^37^ results in acceptable knee function,^7^ and restores passive anterior-posterior stability,^11^ but it does not necessarily prevent subsequent degenerative sequelae,^32,55^ as many patients develop early knee osteoarthritis (OA).^51,52^ Knee health outcomes are particularly poor if meniscal injury is sustained concurrent to ACL rupture,^58^ constituting a 3.5-fold increase in risk of future knee OA compared with ACLR with intact menisci.^22^ However, the mechanism or mechanisms behind this elevated risk are unclear.

After ACLR, patients walk with a straighter knee^67^ and a more externally rotated tibia,^65^ while experiencing smaller external knee flexion moments^67^ that are supported by reduced quadriceps activation.^12^ Autograft harvesting from the semitendinosus and gracilis muscles may result in subsequent atrophy^44^ and the commonly observed knee flexion^5,44^ and internal^6,44^ rotation strength deficits. Given the medial locations of the semitendinosus and gracilis muscles, their varus moment arms about the knee,^13^ and their contributions to medial tibiofemoral contact forces,^63,74^ it is possible that the reported reductions in the medial tibiofemoral contact forces following ACLR^63,72^ are partly due to donor muscle impairment.

Mechanical loading is essential to healthy development and maintenance of articular tissues.^9^ The external knee adduction moment during walking tends to compress the medial tibiofemoral compartment^2^ and has been associated with thicker medial-to-lateral tibiofemoral cartilage in healthy knees,^3,45,46^ but in OA knees it has been associated with thinner cartilage.^3,4,45^ During the first year following ACLR, humans lose ∼10% of tibial bone mineral density^56^; 2 weeks after induced ACL injury combined with subsequent mechanical unloading, mice lose ∼50% of their distal femoral trabecular bone.^1^ In humans, those who developed radiographic medial knee OA by 5 years post-ACLR had, compared with their intact contralateral knee, ∼25% smaller medial tibiofemoral contact forces 6 months post-ACLR.^72^ Together, these studies suggest that lower-than-normal knee loading shortly after ACL injury is a factor in future knee OA development. In human ACLR knees, the relationships between the tibiofemoral contact forces produced during common daily activities (eg, walking) and cartilage morphology remain unexplored.

The purpose of this study was (1) to examine the relationships between the tibiofemoral contact forces generated during walking and tibial articular cartilage morphology in knees 2 to 3 years following ACLR, and (2) to assess if meniscal injury influenced these relationships. We focused on walking instead of vigorous gait tasks such as running because walking is the most common form of human ambulation and, thus, the main behavioral determinant of the knee’s habitual mechanical environment. Through advances in computational modeling, it is now possible to predict tibiofemoral contact forces in real time and to use these contact forces as biofeedback to modulate gait biomechanics.^60^ This enables clinicians to directly target tibiofemoral contact forces during gait rehabilitation following ACLR, potentially influencing cartilage morphology. However, we must first understand the relationships between tibiofemoral contact forces and cartilage morphology in ACLR knees. We hypothesized that in meniscal-injured ACLR knees, greater contact forces would be related to smaller cartilage volumes and thicknesses, reflective of the early knee degeneration that is common after ACLR with concurrent meniscal pathology^22,58^ and similar to the relationships reported in OA knees.^33,34,42^ Conversely, those with intact menisci should have minimal knee degeneration at 2 to 3 years post-ACLR, as indicated by their relatively low rates of knee OA compared with meniscal-injured ACLR patients.^47,57^ Therefore, in isolated ACLR knees, we hypothesized greater contact forces would be related to greater cartilage volumes and thicknesses, similar to the positive relationships reported in healthy knees.^3,4^

## Methods

This cross-sectional case-control study was conducted with institutional human research ethics approval, and participants provided their informed written consent prior to testing. A total of 100 individuals with ACLR (age at testing, 29.7 ± 6.5 years) were recruited from the clinic records of private consultancies, and 30 healthy individuals (age at testing, 28.3 ± 5.2 years) were recruited by word of mouth from the local communities to serve as controls. Inclusion criteria for all participants included body mass index ≤34 kg/m^2^, age 18 to 42 years, no neuromusculoskeletal or cardiovascular condition, and no self- or clinician-diagnosed OA anywhere in the body. Inclusion criteria specific to ACLR participants at time of testing included 2 to 3 years following ipsilateral single-bundle combined semitendinosus and gracilis ACLR, ≤6 months between ACL rupture and ACLR, no revisions, and no contralateral ACL rupture or ACLR; in addition, any cartilage lesions identified during surgery had to have an International Cartilage Repair Society grade of ≤1. We did not include previous grade of sports participation and physical activity levels at time of testing or preinjury as inclusion or exclusion criteria.

Reconstructions were performed by 4 fellowship-trained orthopaedic surgeons. All procedures were arthroscopically assisted, with full-length tunnel outside-in tibial drilling followed by either transtibial or anteromedial portal drilling of the femoral tunnel. Irrespective of femoral drilling technique, the aim was to place the femoral tunnel within the anteromedial portion of the native ACL femoral footprint. Semitendinosus and gracilis tendons were harvested via a 3- to 4-cm incision over the pes anserinus, and a 4-strand autograft construct was created. Femoral fixation was undertaken with a closed-loop Endobutton (Smith & Nephew Endoscopy) and tibial fixation by an interference screw. Meniscal repair was performed if the surgeon deemed a lesion repairable; otherwise, it was resected or, if stable, left untreated.

From the overall cohort of ACLR participants, 62 were grouped “isolated ACLR” (age at testing, 29.8 ± 6.4 years; weight, 74.9 ± 13.3 kg) as they had no meniscal pathology at time of surgery, and 38 were grouped “meniscal-injured ACLR” (age at testing, 30.6 ± 6.6 years; weight, 83.3 ± 14.3 kg). In the meniscal-injured group, we included those with repaired, resected, and untreated but stable meniscal pathology. Our rationale for grouping these different types of meniscal injury was that, in all cases, the load-distributing function of the menisci would be impaired in some way, and this would likely influence the relationships between contact forces and cartilage morphology. Meniscal extrusion was not assessed.

Within 1 day after ACLR, participants were discharged from the hospital, instructed to return to full weightbearing, and permitted unrestricted knee range of motion. Participants immediately enrolled in criterion-based physical therapy,^66^ which aimed to restore the ACLR knee’s passive and dynamic range of motion as well as muscle strength. Particular emphasis was placed on vastus medialis recruitment, as retention of vastus medialis cross-sectional area has been associated with reduced rates of medial knee cartilage loss and better clinical outcomes.^71^ Participants were instructed to begin training using a stationary bicycle by week 4, were graduated to vigorous straight-line running by 3 to 4 months, and commenced sport-specific exercises at 4 months. When participants could complete prescribed exercises without pain or swelling, they were graduated to more challenging tasks.

Magnetic resonance imaging (MRI) was performed on ACLR knees and randomized knees from the controls. We did not compare ACLR with contralateral knees because abnormal loading of the contralateral knee in ACLR patients^75^ limits use as a control. A 1.5T Signa (GE Healthcare) or 3T Megenetom Verio (Siemens) MR unit was used. Sagittal images were acquired using T1-weighted fat-suppressed 3-dimensional gradient recall sequences in steady state with either 55° flip angle, 44 ms repetition time, 12 ms echo time, 16 cm field of view, 1.5 cm slice thickness, 60 partitions, and 256 × 256 matrix, or 10° flip angle, 12.5 ms repetition time, 4.9 ms echo time, 16 cm field of view, 1.5 cm slice thickness, 60 partitions, and 512 × 512 matrix. All coronal images used proton density–weighted fat-saturated spin echo sequences, with a 3500 to 3800 ms repetition time, 50 ms echo time, 13 cm field of view, 3 mm slice thickness, and 1 mm interslice gap.

The MRIs were used to measure tibial articular cartilage volumes^59^ and the underlying bone plate areas,^19^ the results of which have been previously published.^70^ Image processing was performed by 1 examiner, blinded to participant grouping and meniscal injury status, using OsiriX medical image processing software.^61^ From each MRI, the tibial cartilage was identified by manually segmenting the tissue boundary using the software’s graphical user interface. Successive segmentations were then interpolated, and volume (mm^3^) was calculated by summing bounded voxels. The same approach was applied to measure tibial bone plate areas (mm^2^), which are the bones immediately underlying the tibial articular cartilage. This method to measure cartilage volumes has been shown to be accurate and reproducible.^19^ The current study used MRI units with equivalent or greater field strength, as well as similar image-acquisition sequences and image-processing methods, to those of the validation study. Measurements of tibial articular cartilage volumes and bone plate areas for the ACLR and control participants had excellent intrarater reliability (ie, intraclass correlation coefficients [ICCs] of >0.99). A random cross-check, performed by an experienced musculoskeletal radiologist, revealed interrater reliability ICCs of >0.98.^70^ Bulk measures of cartilage thickness (mm) were calculated by dividing compartmental cartilage volumes by underlying bone plate areas.

Each participant underwent laboratory-based gait analysis <1 week after MRI, the details of which are published elsewhere.^63^ To summarize, participants walked over ground at their self-selected pace, while 3-dimensional body motion, ground-reaction forces, and surface electromyography (EMG) were concurrently and synchronously acquired. Surface EMG was performed on 8 major knee muscles from the MRI leg: medial and lateral gastrocnemius, hamstrings, and vasti, as well as rectus femoris and tensor fasciae latae. Walking biomechanics, determined using OpenSim^26^ version 3.2, were then used, along with the EMG, to calibrate and execute an EMG-driven model of muscle^14,50^ and tibiofemoral contact^64,74^ forces (N). For ACLR participants, the model semitendinosus was modified to account for morphologic changes after autograft harvesting,^73^ as previously described.^63^ Gait biomechanics and tibiofemoral contact forces were time normalized to 100% of gait cycle. For each participant, maximum tibiofemoral contact forces during the stance phase were averaged across 3 repeated trials, and these averaged values were used in subsequent statistical analyses.

Statistical analyses were performed using SPSS (v 22; IBM Corp). Participant demographics, anthropometrics, cartilage thicknesses, and gait spatiotemporal parameters were assessed using chi-square or Student *t* tests for nonparametric and parametric data, respectively. General linear models were used to assess the relationships between contact forces and cartilage morphology (ie, volumes and thicknesses). To assess the influence of meniscal injury, we identified those who had medial or lateral meniscal injury at the time of arthroscopy and then regressed cartilage morphology from the injured compartment onto the corresponding contact forces. Those with meniscal injury in both compartments were included in both regressions.

## Results

The ACLR and the control participants had similar demographics, anthropometrics, and gait spatiotemporal parameters (Table 1), but meniscal-injured ACLR participants had significantly greater body mass (83.3 ± 14.3 kg) and body mass index (26.9 ± 4.1 kg/m^2^) compared with both isolated ACLR (74.9 ± 13.3 kg and 24.2 ± 2.8 kg/m^2^, respectively) and control (74.9 ± 14.9 kg and 23.4 ± 3.3 kg/m^2^, respectively) participants. Times from injury to ACLR and from ACLR to testing were not significantly different between the 2 ACLR groups (Table 1).

**TABLE 1:** Characteristics and Gait Spatiotemporal Parameters of the Controls and of the Entire Cohort of ACLR, Isolated ACLR, and Meniscal-Injured ACLR Participants*^a^*

	Controls (N = 30)	All ACLR (N = 100)	Isolated ACLR (n = 62)	Meniscal-Injured ACLR (n = 38)
Males, n (%)	19 (63)	66 (66)	42 (68)	24 (63)
Age, y	28.3 ± 5.2	29.7 ± 6.5	29.8 ± 6.4	30.6 ± 6.6
Mass, kg	74.9 ± 14.9	78.1 ± 14.4	74.9 ± 13.3	83.3 ± 14.3*^b,c^*
Body mass index, kg/m^2^	23.4 ± 3.3	25.2 ± 3.6*^b^*	24.2 ± 2.8*^b^*	26.9 ± 4.1*^b,c^*
Height, m	1.79 ± 0.09	1.76 ± 0.08	1.75 ± 0.09	1.76 ± 0.06
Injury to surgery, y	NA	0.21 ± 0.14	0.20 ± 0.11	0.24 ± 0.17
Right knees tested, n (%)	13 (43)	51 (51)	32 (52)	17 (44)
Surgery to testing, y	NA	2.51 ± 0.44	2.5 ± 0.4	2.6 ± 0.5
Walking speed, m/s	1.44 ± 0.22	1.41 ± 0.18	1.42 ± 0.2	1.42 ± 0.19
Stride length, m	1.51 ± 0.12	1.51 ± 0.10	1.50 ± 0.1	1.52 ± 0.11
Stride time, s	1.08 ± 0.09	1.11 ± 0.06	1.11 ± 0.06	1.1 ± 0.05
Stride rate, strides/min	0.93 ± 0.074	0.91 ± 0.05	0.91 ± 0.05	0.91 ± 0.04

*^a^*Data are reported as mean ± SD unless otherwise indicated. ACLR, anterior cruciate ligament reconstruction; NA, not applicable.

*^b^*Significantly different from controls, *P* < .05.

*^c^*Significantly different from isolated ACLR, *P* < .05.

As previously reported,^70^ isolated ACLR knees had smaller medial cartilage volumes (2164.1 ± 651 mm^3^) compared with those of controls (2513.9 ± 691 mm^3^) and similar values to those of meniscal-injured (2213.7 ± 595 mm^3^) knees. No differences in lateral cartilage volumes, nor any differences in bone plate areas, were found among control, isolated, or meniscal-injured ACLR knees (Table 2). Control knees had thicker medial and lateral cartilage compared with that of isolated and meniscal-injured ACLR knees, while no significant differences in cartilage thickness were found between isolated and meniscal-injured ACLR knees (Table 3).

**TABLE 2:** Tibial Cartilage Volumes and Bone Plate Areas From the Knees of the Controls and From the Entire Cohort of ACLR, Isolated ACLR, and Meniscal-Injured ACLR Knees*^a^*

	Tibial Compartment	Controls (N = 30)	All ACLR (N = 100)	Isolated ACLR (n = 62)	Meniscal-Injured ACLR (n = 38)
Cartilage volume, mm^3^	Medial	2513.9 ± 691	2182.9 ± 628*^b^*	2164.1 ± 651*^b^*	2213.7 ± 595
	Lateral	3145.4 ± 880	2905.1 ± 824	2920.8 ± 846	2879.6 ± 797
Bone plate area, mm^2^	Medial	2289.1 ± 357	2262.4 ± 325	2222.6 ± 335	2327.4 ± 300
	Lateral	1296.5 ± 222	1304 ± 200	1313 ± 209	1289.2 ± 186

*^a^*These data (reported as mean ± SD) were previously reported by Wang et al^70^ and are presented here for convenience. ACLR, anterior cruciate ligament reconstruction.

*^b^*Significantly different from the controls, *P* < .05.

**TABLE 3:** Tibial Cartilage Thickness in the Medial and Lateral Compartments of Control Knees and in the Entire Cohort of ACLR, Isolated ACLR, and Meniscal-Injured ACLR Knees*^a^*

	Mean Tibial Cartilage Thickness, mm	
	Medial	Lateral
Controls	1.09 ± 0.23	2.41 ± 0.08
All ACLR	0.93 ± 0.18*^b^*	2.15 ± 0.04*^b^*
Isolated ACLR	0.92 ± 0.17*^b^*	2.15 ± 0.37*^b^*
Meniscal-injured ACLR	0.94 ± 0.20*^b^*	2.20 ± 0.47*^b^*

*^a^*Data are reported as mean ± SD. ACLR, anterior cruciate ligament reconstruction.

*^b^*Significantly different from healthy controls, *P* < .05.

**Figure 1.**
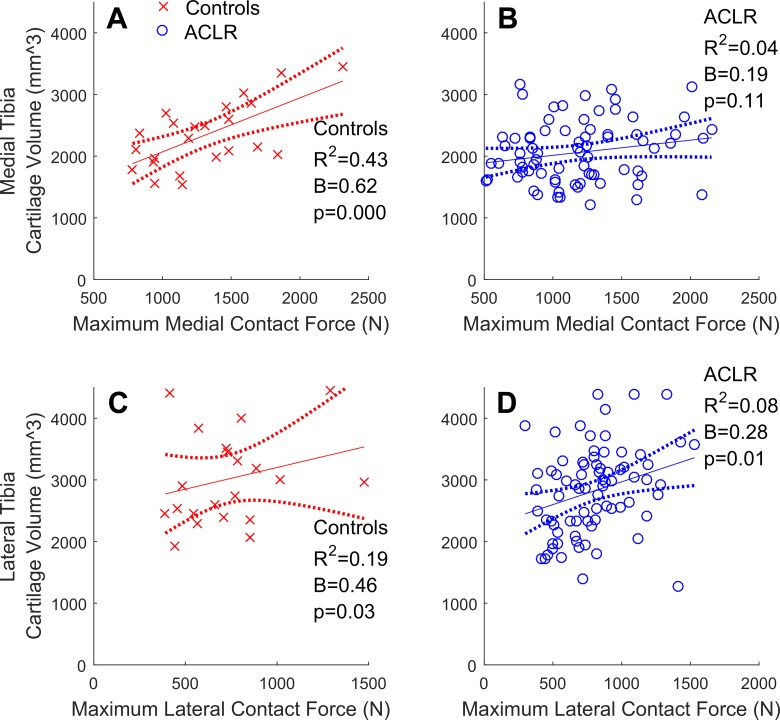
Tibial articular cartilage volumes (mm^3^) regressed onto maximum walking tibiofemoral contact forces (N) with the 95% confidence intervals (dotted lines) for the control (cross-hairs) and overall cohort of ACLR knees (circles). (A and B) Medial compartment; (C and D) lateral compartment. ACLR, anterior cruciate ligament reconstruction.

**Figure 2.**
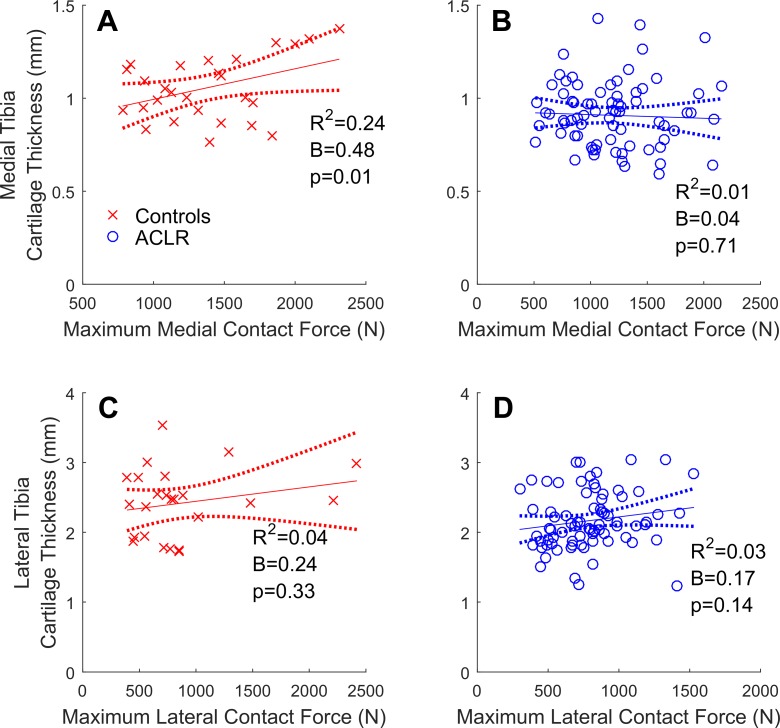
Tibial articular cartilage thicknesses (mm) regressed onto maximum walking tibiofemoral contact forces (N) with the 95% confidence intervals (dotted lines) for the control (cross-hairs) and overall cohort of ACLR knees (circles). (A and B) Medial compartment; (C and D) lateral compartment. ACLR, anterior cruciate ligament reconstruction.

In both compartments of control knees, greater contact forces were significantly related to greater cartilage volumes (Figure 1, A and C). Similarly, significant positive relationships were found in the lateral compartment of the overall cohort of ACLR knees (Figure 1D) but not in the medial compartment (Figure 1B), where the relationship was nonsignificant and flat. In control knees, greater contact forces were related to thicker medial, but not lateral, cartilage (Figure 2, A vs C). In the overall cohort of ACLR knees, no significant relationships between contact forces and cartilage thicknesses were found (Figure 2, B and D).

**Figure 3.**
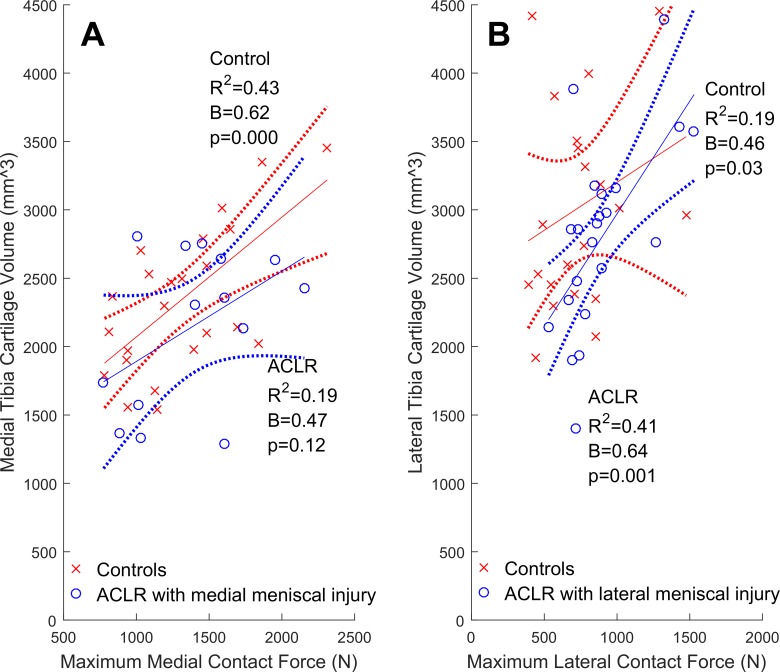
Tibial articular cartilage volumes (mm^3^) from the compartment that sustained a meniscal injury regressed onto maximum contact forces (N) with 95% confidence intervals (dotted lines). Shown are ACLR participants (circles) with (A) medial meniscal injury (n = 17) and (B) lateral meniscal injury (n = 25), while healthy controls (cross-hairs) are plotted for comparision. ACLR, anterior cruciate ligament reconstruction.

**Figure 4.**
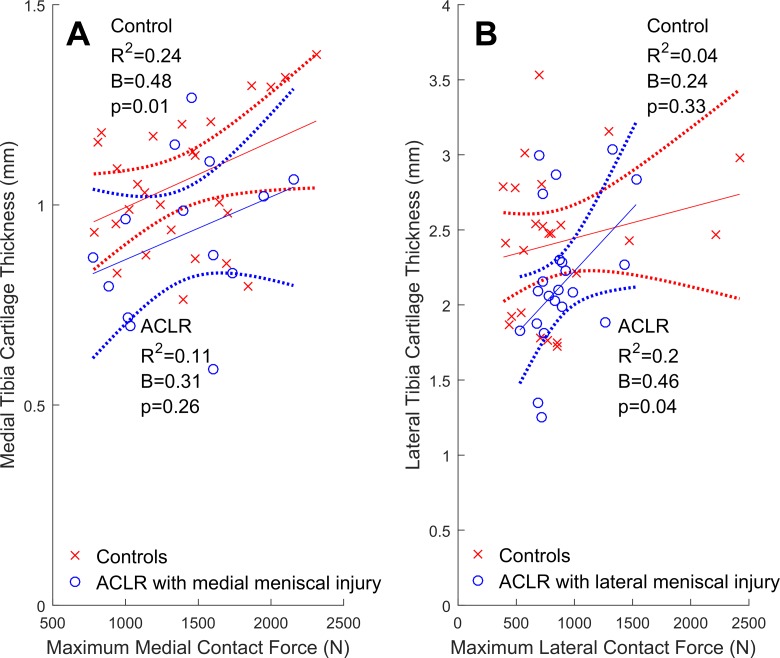
Tibial articular cartilage thickness (mm) from the compartment that sustained a meniscal injury regressed onto maximum contact forces (N) from the respective tibiofemoral compartment with 95% confidence intervals (dotted lines). Shown are ACLR participants (circles) with (A) medial meniscal injury (n = 17) and (B) lateral meniscal injury (n = 25), while healthy controls (cross-hairs) are plotted for comparision. ACLR, anterior cruciate ligament reconstruction.

**Figure 5.**
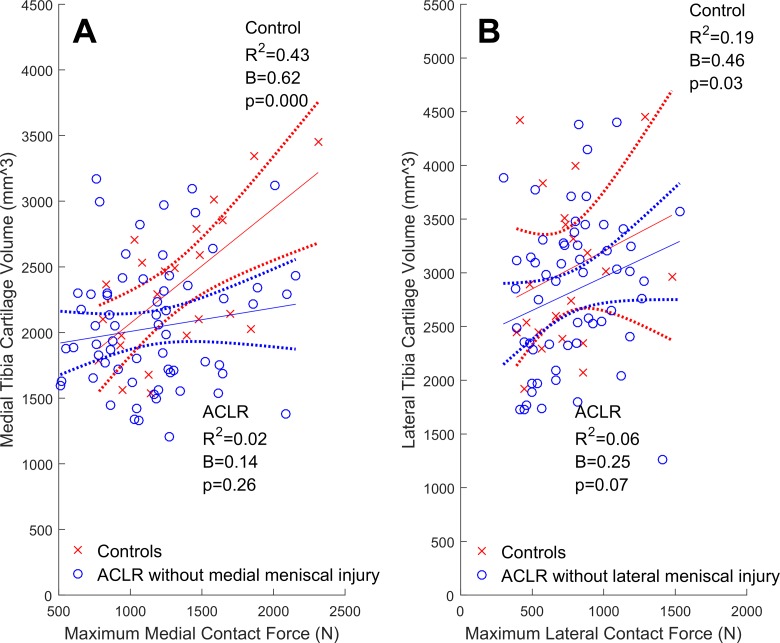
Tibial articular cartilage volumes (mm^3^) regressed onto maximum contact forces (N) with 95% confidence intervals (dotted lines) for isolated ACLR and healthy control knees in the (A) medial and (B) lateral compartments. ACLR, anterior cruciate ligament reconstruction.

**Figure 6.**
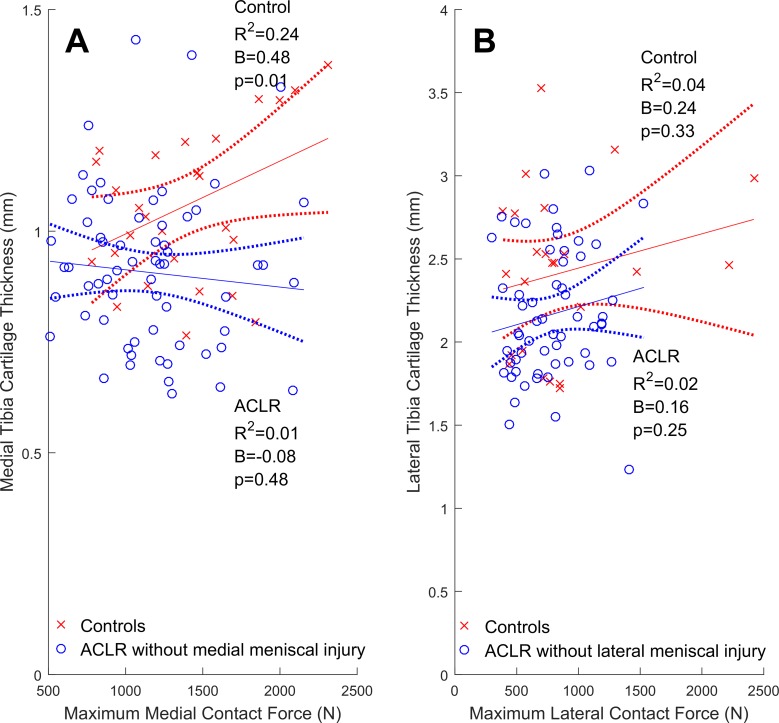
Tibial articular cartilage thickness (mm) regressed onto maximum contact forces (N) with 95% confidence intervals (dotted lines) for isolated ACLR and healthy control knees in the (A) medial and (B) lateral compartments. ACLR, anterior cruciate ligament reconstruction.

The types of meniscus injuries sustained are listed in Table 4. The lateral meniscus was most commonly affected (n = 25, ∼66% of meniscal-injured ACLR knees), which included 12 cases of partial resection with no other meniscal injury or treatment. The remaining cases (n = 12, 36%) were combinations of untreated meniscal tears, repairs, and resections in either or both compartments. In ACLR knees with lateral meniscal injury, greater lateral contact forces were significantly related to greater lateral cartilage volumes and thickness (Figure 3B and Figure 4B). In contrast, ACLR knees with medial meniscal injury had relationships between medial contact forces and both medial cartilage volumes (Figure 3A) and thicknesses (Figure 4A) that were nonsignificant (*P* = .12 and .26, respectively). In both the medial and lateral compartments of isolated ACLR knees, we did not find significant relationships between contact forces and either cartilage volumes (Figure 5) or thicknesses (Figure 6).

**TABLE 4:** Prevalence of Different Types of Meniscal-Injured ACLR Knees*^a^*

	Prevalence	
Meniscal Injury Type	n	%
Untreated medial tear	4	11
Untreated lateral tear	6	16
Medial resection	5	13
Lateral resection	12	32
Untreated medial tear and lateral resection	2	5
Medial and lateral resections	1	3
Medial resection and lateral repair	1	3
Medial repair and untreated medial tear	4	11
Lateral repair and untreated lateral tear	3	8

*^a^*Rounding errors and some participants having multiple meniscal injuries resulted in the sum of injury type percentages equaling more than 100%. ACLR, anterior cruciate ligament reconstruction.

## Discussion

To our knowledge, this study was the first to investigate the relationships between tibiofemoral contact forces and tibial articular cartilage morphology in ACLR knees and to explore the influence of meniscal injury on these relationships. In healthy control knees, greater contact forces were related to greater cartilage volumes in both tibiofemoral compartments and to thicker medial, but not lateral, cartilage. In the overall cohort of ACLR knees, greater contact forces were related to greater cartilage volumes in the lateral, but not medial, compartment and had no significant relationships to cartilage thicknesses in either compartment. However, in lateral meniscal-injured ACLR knees, greater lateral contact forces were significantly related to greater lateral cartilage volumes and thicknesses; in medial meniscal-injured ACLR knees, no significant relationships between contact force and cartilage morphology were found. Follow-up investigation should examine if the relationships found at 2 to 3 years post-ACLR are associated with future knee health.

In healthy control knees, greater contact forces were related to greater cartilage volumes in both the medial ( Figure 1A) and lateral (Figure 1C) compartments, while greater contact forces were related to thicker medial (Figure 2A), but not lateral (Figure 2C), cartilage. The results pertaining to the medial compartment were consistent with previous reports,^3,4,45,46^ which found that greater knee adduction moments (ie, a surrogate of medial-to-lateral tibiofemoral contact forces) correlated (0.31 < *R* ^2^ < 0.63) with greater medial-to-lateral tibiofemoral cartilage thickness ratios. A recent study using a computational model to estimate tibiofemoral cartilage contact pressures also found moderate correlations (0.23 < *R* ^2^ < 0.61) with cartilage thickness in healthy knees.^69^ A plausible interpretation of our results as well as those of earlier studies^3,4,45,46,69^ is that healthy knee cartilage thickens in response to the repetitive high-magnitude contact loads experienced during daily activities such as walking. This interpretation is appealing, as it conforms to the fundamentals of cartilage mechanobiology^15–17,41^ and is consistent with both human^18,40^ and animal^42,43^ studies examining the response of cartilage to loading, but it neglects several important observations. First, lateral tibial cartilage is thicker compared with medial cartilage (Table 2 and previous reports^21,28^), despite the lateral compartment experiencing substantially smaller contact forces during walking (ie, 30%-60% of medial values).^31^ If we accept that cartilage optimizes morphology based on loading, as indicated by a wealth of mechanobiology studies, then why are regions of cartilage that sustain smaller contact forces (eg, the lateral tibia) thicker than those that experience substantially higher loading? Second, the reported relationships between contact loads and healthy cartilage morphology range from weak to moderate (ie, 0.19 < *R* ^2^ < 0.63), and in our study, we did not find any significant relationships in the lateral compartment (Figure 2B). Overall, these observations indicate that factors other than contact force magnitudes must also modulate cartilage morphology, particularly in the lateral compartment. Cartilage contact pressure is potentially one such factor, as it incorporates both contact force magnitude and the local tissue area over which the forces are applied. Van Rossom et al^69^ reported significant correlations between cartilage contact pressures and healthy cartilage thicknesses similar to our results (ie, 0.22 < *R* ^2^ < 0.50), and notably they found significant relationships in the lateral compartment. This suggests that using contact pressures may reveal cartilage load–morphology relationships that would remain undetected using contact forces alone. Importantly, both contact forces and pressures are applied loads and do not directly account for the responses of the tissue (ie, strains), which have been recognized as critical to tissue remodeling in engineered cartilage^48^ and cause bone remodeling.^33^ An analysis of the strains experienced by ACLR tibiofemoral cartilage during walking is warranted but was beyond the scope of this study.

In the overall cohort of ACLR knees, relationships between contact forces and cartilage thicknesses were found to be flat and nonsignificant (Figure 2, B and D). They appeared to be somewhere between the positive relationships reported from healthy knees (Figure 2, A and C, and previous reports^2,3,32,69^) and negative relationships from OA knees.^3,4,45^ It may be that, in the overall cohort of ACLR knees, degenerative changes had already begun, but without follow-up data, we cannot comment on whether the relationships of contact force to cartilage morphology seen 2 to 3 years post-ACLR are associated with future knee health. An implication of these flat relationships is that the largest-magnitude contact forces were not sustained by the thickest cartilage, potentially placing the tissue at risk of injury and degeneration. The medial and lateral tibial cartilage in the overall cohort of ACLR knees was 0.16 and 0.36 mm thinner, respectively, compared with the healthy control knees (Table 3). If we assume that, prior to surgery, the ACLR participants had cartilage of similar thickness to that of healthy control knees, then these differences amount to ∼15% and 11% of healthy medial and lateral cartilage, respectively. If we further assume that the cartilage losses were linearly distributed from time of ACLR to study participation, this constituted ∼3% to 5% annual loss of cartilage thickness. In knee OA patients, a similar 3% to 5% annual loss of cartilage was associated with significant risk (ie, odds ratio, 2.3; 95% confidence interval, 0.4-12.2) for knee arthroplasty within 4 years.^20^ This does not mean the ACLR participants in this study are likely to receive knee replacement within 4 years, as they had thicker and better cartilage to begin with compared to knee OA patients. If their rate of cartilage loss remains unabated, it will lead to knee failure.

Previously, it has been reported that cartilage defects were more prevalent in the meniscal-injured than in isolated ACLR knees.^70^ These initial results motivated us to examine the relationships between contact forces and cartilage morphology, as well as the potential influence of meniscal injury on these relationships. We found that partial lateral meniscus resection was the most common form of meniscal injury in the ACLR participants (Table 4), which is consistent with both arthroscopic reports of acute ACL injuries^38^ and the observed valgus collapse of the knee that commonly occurs during ACL rupture.^23^ Contrary to our primary hypothesis, we found that greater contact forces were related to greater cartilage volumes and thicknesses in the lateral compartment of lateral meniscal-injured ACLR knees but not in the medial compartment of medial meniscal-injured ACLR knees. This explained why the relationships between lateral contact forces and lateral cartilage volumes in the overall cohort of ACLR knees were found to be significant and positive (Figure 1D) while nonsignificant in the medial compartment (Figure 1B). However, these positive relationships in lateral meniscal-injured ACLR knees may not be indicative of functional adaptations of the cartilage to the habitual loading environment and may be due to fluctuations in cartilage morphology following joint trauma (eg, ACL rupture and ACLR). Indeed, within 5 years of ACL injury, tibiofemoral cartilage has been shown to both swell and thin, sometimes within the same compartment.^29^ Moreover, this swollen cartilage may be of poorer quality (ie, increased T_2_ and T_1*p*_ relaxation times reported at 2 years^68^ and 3 years^8^ post-ACLR). In the current study, we did not assess cartilage quality, but we noted that significant positive relationships between contact forces and cartilage morphology were observed in the common lateral meniscal-injured ACLR knees but were not seen in medial meniscal-injured ACLR knees. We have interpreted this to mean that the compartment where meniscal injury is sustained, and not simply the presence or absence of a meniscal injury, influences the relationships between contact forces and cartilage morphology. Future studies may consider examining whether other features of meniscal injury, such as size and type, have unique influences on the relationships between contact force and cartilage morphology.

In contrast to meniscal-injured ACLR knees and contrary to our hypothesis, we found nonsignificant relationships between tibiofemoral contact forces and both cartilage volumes (Figure 5) and thicknesses (Figure 6) in isolated ACLR knees. These results may mean that osteoarthritic changes have already begun by ∼3 years following the initial ACL injury, as we did not find the significant positive relationships between contact forces and cartilage morphology characteristic of healthy control knees. This result, coupled with the rate of cartilage loss thought to have occurred in the ACLR knees, do not auger well for future knee health. However, we tested these ACLR participants only at a single time point (ie, 2-3 years postsurgery), and both cartilage morphology^29^ and tibiofemoral contact force magnitudes^72^ are known to fluctuate considerably over the first 5 years after ACL injury. Thus, it is possible the relationships we found were transient or peculiar to the 2- to 3-year postoperative time period. This leaves open the possibility that these relationships may change in time to match those seen in healthy knees, as has been shown to occur with the tibiofemoral contact forces in ACLR knees.^72^ A prospective study examining the progression of these relationships throughout ACLR rehabilitation and the first 5 years that follow would be insightful for both researchers and clinicians alike.

We found no significant differences in cartilage thicknesses between isolated and meniscal-injured ACLR knees, which was consistent with a previous study^70^ that reported no differences in cartilage volumes, nor underlying bone plate areas, between these 2 ACLR groups. Similarly, Lee et al^49^ found that meniscal injury did not affect semiquantitative assessment of cartilage health at ∼2 years post-ACLR follow-up, and Wellsandt et al^72^ reported no differences in prevalence of meniscal injury between those who did and did not develop radiographic knee OA 5 years post-ACLR. In these studies,^49,70,72^ the time from surgery to evaluation of knee health was 2 to 5 years, which may not be sufficient time for MRI-based measurements or clinical assessments to detect degeneration. In general, reports of equivalent knee health outcomes in isolated and meniscal-injured ACLR depart from the consensus opinion that meniscal injury is a particularly potent risk factor for future knee OA onset.^24,30,51–53,62^ In cases of nonsurgically treated damaged menisci^30^ and in the majority of meniscal-injured ACLR studies,^62^ patients were aged 61.6 ± 7.9 years and ∼40 years, respectively, compared with the younger participants in this study (29.7 ± 6.5 years). This is important because radiographic signs of degeneration following meniscal injury are worse in older compared with younger knees.^10^ Further, in the cited studies,^19,25,39–41,51^ patients were assessed considerably later compared with our study (ie, >10 years vs 2-3 years after injury), which provided time for meniscal injury to influence cartilage morphology and for the aging process to affect cartilage. In our younger cohort of ACLR participants, cartilage degeneration due to meniscal injury may not emerge until several years have passed. However, given the weight of evidence indicating that meniscal injury is a potent risk factor for knee OA, our findings of equivalent cartilage thickness between isolated and meniscal-injured ACLR knees at 2 to 3 years postsurgery should be interpreted cautiously.

Several limitations of this study should be considered. First, we assumed that the relationships between contact forces and cartilage morphology were linear and, thus, amenable to general linear models. This is an established approach in the literature, but it may not be appropriate given that cartilage is a complex biologic tissue with remodeling processes that are both nonlinear and dynamic. An alternative to the current approach would be to couple our neuromusculoskeletal model estimates of contact forces with a mechanistic model of cartilage remodeling.^34^ Second, we know that forces applied to tissues such as cartilage will cause strains, and these may stimulate remodeling processes. However, we could not determine causal relationships between the tibiofemoral contact forces and cartilage morphologies due to the simple cross-sectional design of this study. For example, in healthy control knees, greater contact forces may have caused the cartilage to adapt by increasing volume, or those with more cartilage volume may simply have had greater contact forces. Therefore, we confined ourselves to reporting the relationships for the different clinical and nonclinical groups in our study and did not posit causal relationships. Third, increased prevalence of articular tissue pathologies, such as cartilage defects, have been reported in the meniscal-injured knee compared with isolated ACLR knees.^70^ This could have resulted in the meniscal-injured participants’ using a gait strategy to unload their damaged knee, ostensibly due to pain. However, there were no significant differences in walking speeds, knee angles, external knee moments, or tibiofemoral contact forces between the isolated and the meniscal-injured ACLR participants,^63^ and they reported similar levels of knee pain.^70^ This means there is no evidence to support the idea that pain-induced gait modifications explain the relationships between contact force and cartilage morphology in the 2 different groups of ACLR participants. Future studies may consider exploring whether pain early in ACLR rehabilitation affects longer-term gait biomechanics and cartilage morphology.

A fourth limitation of our study was that in the meniscal-injured ACLR group, we included those with single and multiple, small and large, repaired and unrepaired, and resected and untouched meniscal injuries. We grouped them together for pragmatic reasons: There were 9 different types of meniscal injury, and we did not have sufficient participant numbers to examine each type independently. Each of these variations on meniscal injury may have had different influences on the relationships between contact forces and cartilage morphology, which may have confounded our analysis when grouped together. Fifth, postoperative physical rehabilitation was not controlled, and we have no data on individual compliance with therapy or when ACLR participants returned to high-loading activities. Some may have returned to high-loading activities earlier compared with others and, hence, had greater exposure to high-loading activities by their time of testing, which may have influenced cartilage morphology, contact forces, and their relationships. Sixth, these data were acquired before current knee-imaging recommendations were published,^39^ which means that the MRIs were not acquired using the current recommended sequences of proton density–weighted, fast/turbo spin echo, or T2-weighted turbo spin echo to image tibiofemoral cartilage. Seventh, we used MRI units of different field strength (ie, 1.5 and 3 T), which may have influenced the cartilage morphology measurements. However, no participant was imaged using both MRI units, so we cannot confirm that the different MRI units influenced our cartilage measurements but acknowledge that it is a potential limitation. Finally, it is not currently possible to measure in vivo contact forces in native human joints. The EMG-driven model used to determine the tibiofemoral contact forces has been indirectly validated, as it has been shown to accurately predict contact forces produced during gait measured through instrumented prosthetic knee implants,^31,36^ as well as the external joint moments determined through inverse dynamics.^14,50^ Although model predictions of the lateral contact forces have been shown to be less accurate^36^ and reliable^35^ than medial contact forces, we are confident that our predictions of the tibiofemoral contact forces were appropriate for the scope of this investigation.

## Conclusion

Isolated and meniscal-injured knees at 2 to 3 years post-ACLR had thinner medial and lateral tibial cartilage than that of healthy control knees. In healthy control knees, greater contact forces were related to greater cartilage volumes in both the medial and lateral compartments, as well as thicker medial cartilage. By 2 to 3 years following surgery, in both the overall cohort and isolated ACLR knees, flat relationships were found between contact forces and cartilage thicknesses, meaning that the largest-magnitude forces were not supported by the thickest cartilage. In lateral meniscal-injured ACLR knees, greater lateral contact forces were related to thicker cartilage, although it was not clear if this was an adaptive response or a swelling that often occurs in the first 5 years after ACLR.

## References

[1] Anderson MJ Diko S Baehr LM Baar K Bodine SC Christiansen BA Contribution of mechanical unloading to trabecular bone loss following non-invasive knee injury in mice. J Orthop Res. 2016;34(10):1680–1687. 26826014 10.1002/jor.23178 PMC5603199

[2] Andriacchi TP Dynamics of knee malalignment. Orthop Clin North Am. 1994;25(3):395–403. 8028883

[3] Andriacchi TP Koo S Scanlan SF Gait mechanics influence healthy cartilage morphology and osteoarthritis of the knee. J Bone Joint Surg Am. 2009;91(suppl 1):95–101. 10.2106/JBJS.H.01408 PMC2663350 19182033

[4] Andriacchi TP Mundermann A Smith RL Alexander EJ Dyrby CO Koo S A framework for the in vivo pathomechanics of osteoarthritis at the knee. Ann Biomed Eng. 2004;32(3):447–457. 15095819 10.1023/b:abme.0000017541.82498.37

[5] Ardern CL Webster KE Taylor NF Feller JA Hamstring strength recovery after hamstring tendon harvest for anterior cruciate ligament reconstruction: a comparison between graft types. Arthroscopy. 2010;26(4):462–469. 20362824 10.1016/j.arthro.2009.08.018

[6] Armour T Forwell L Litchfield R Kirkley A Amendola N Fowler PJ Isokinetic evaluation of internal/external tibial rotation strength after the use of hamstring tendons for anterior cruciate ligament reconstruction. Am J Sports Med. 2004;32(7):1639–1643. 15494327 10.1177/0363546504263405

[7] Bach BR Jr Tradonsky S Bojchuk J Levy ME Bush-Joseph CA Khan NH Arthroscopically assisted anterior cruciate ligament reconstruction using patellar tendon autograft. Five- to nine-year follow-up evaluation. Am J Sports Med. 1998;26(1):20–29. 9474397 10.1177/03635465980260012101

[8] Bae JH Hosseini A Wang Y Articular cartilage of the knee 3 years after ACL reconstruction. A quantitative T2 relaxometry analysis of 10 knees. Acta Orthop. 2015;86(5):605–610. 25854533 10.3109/17453674.2015.1039426 PMC4564784

[9] Beaupre GS Stevens SS Carter DR Mechanobiology in the development, maintenance, and degeneration of articular cartilage. J Rehabil Res Dev. 2000;37(2):145–151. 10850820

[10] Bolano LE Grana WA Isolated arthroscopic partial meniscectomy. Functional radiographic evaluation at five years. Am J Sports Med. 1993;21(3):432–437. 8346759 10.1177/036354659302100318

[11] Brandsson S Karlsson J Sward L Kartus J Eriksson BI Karrholm J Kinematics and laxity of the knee joint after anterior cruciate ligament reconstruction: pre- and postoperative radiostereometric studies. Am J Sports Med. 2002;30(3):361–367. 12016076 10.1177/03635465020300031001

[12] Bryant AL Kelly J Hohmann E Neuromuscular adaptations and correlates of knee functionality following ACL reconstruction. J Orthop Res. 2008;26(1):126–135. 17676614 10.1002/jor.20472

[13] Buchanan TS Kim AW Lloyd DG Selective muscle activation following rapid varus/valgus perturbations at the knee. Med Sci Sports Exerc. 1996;28(7):870–876. 8832541 10.1097/00005768-199607000-00014

[14] Buchanan TS Lloyd DG Manal K Besier TF Estimation of muscle forces and joint moments using a forward-inverse dynamics model. Med Sci Sports Exerc. 2005;37(11):1911–1916. 16286861 10.1249/01.mss.0000176684.24008.6f

[15] Carter DR Wong M Mechanical stresses and endochondral ossification in the chondroepiphysis. J Orthop Res. 1988;6(1):148–154. 3334736 10.1002/jor.1100060120

[16] Carter DR Wong M Mechanical stresses in joint morphogenesis and maintenance In: Mow VC Ratcliffe A Woo S, eds. Biomechanics of Diarthrodial Joints. Vol 2 New York: Springer-Verlag; 1990:155–174.

[17] Carter DR Wong M The role of mechanical loading histories in the development of diarthrodial joints. J Orthop Res. 1988;6(6):804–816. 3171761 10.1002/jor.1100060604

[18] Chaudhari AM Briant PL Bevill SL Koo S Andriacchi TP Knee kinematics, cartilage morphology, and osteoarthritis after ACL injury. Med Sci Sports Exerc. 2008;40(2):215–222. 18202582 10.1249/mss.0b013e31815cbb0e

[19] Cicuttini F Forbes A Morris K Darling S Bailey M Stuckey S Gender differences in knee cartilage volume as measured by magnetic resonance imaging. Osteoarthritis Cartilage. 1999;7(3):265–271. 10329301 10.1053/joca.1998.0200

[20] Cicuttini FM Jones G Forbes A Wluka AE Rate of cartilage loss at two years predicts subsequent total knee arthroplasty: a prospective study. Ann Rheum Dis. 2004;63(9):1124–1127. 15115714 10.1136/ard.2004.021253 PMC1755122

[21] Cicuttini FM Wluka AE Wang Y Davis SR Hankin J Ebeling P Compartment differences in knee cartilage volume in healthy adults. J Rheumatol. 2002;29(3):554–556. 11908572

[22] Claes S Hermie L Verdonk R Bellemans J Verdonk P Is osteoarthritis an inevitable consequence of anterior cruciate ligament reconstruction? A meta-analysis. Knee Surg Sports Traumatol Arthrosc. 2013;21(9):1967–1976. 23100047 10.1007/s00167-012-2251-8

[23] Cochrane JL Lloyd DG Buttfield A Seward H McGivern J Characteristics of anterior cruciate ligament injuries in Australian football. J Sci Med Sport. 2007;10(2):96–104. 16807104 10.1016/j.jsams.2006.05.015

[24] Cohen M Amaro JT Ejnisman B Anterior cruciate ligament reconstruction after 10 to 15 years: association between meniscectomy and osteoarthrosis. Arthroscopy. 2007;23(6):629–634. 17560477 10.1016/j.arthro.2007.03.094

[25] Cumps E Verhagen E Annemans L Meeusen R Injury rate and socioeconomic costs resulting from sports injuries in Flanders: data derived from sports insurance statistics 2003. Br J Sports Med. 2008;42(9):767–772. 18048438 10.1136/bjsm.2007.037937

[26] Delp SL Anderson FC Arnold AS OpenSim: open-source software to create and analyze dynamic simulations of movement. IEEE Trans Biomed Eng. 2007;54(11):1940–1950. 18018689 10.1109/TBME.2007.901024

[27] Dunn WR Lincoln AE Hinton RY Smith GS Amoroso PJ Occupational disability after hospitalization for the treatment of an injury of the anterior cruciate ligament. J Bone Joint Surg Am. 2003;85-A(9):1656–1666. 12954822 10.2106/00004623-200309000-00002

[28] Eckstein F Winzheimer M Hohe J Englmeier KH Reiser M Interindividual variability and correlation among morphological parameters of knee joint cartilage plates: analysis with three-dimensional MR imaging. Osteoarthritis Cartilage. 2001;9(2):101–111. 11237657 10.1053/joca.2000.0365

[29] Eckstein F Wirth W Lohmander LS Hudelmaier MI Frobell RB Five-year followup of knee joint cartilage thickness changes after acute rupture of the anterior cruciate ligament. Arthritis Rheumatol. 2015;67(1):152–161. 25252019 10.1002/art.38881

[30] Englund M Guermazi A Roemer FW Meniscal tear in knees without surgery and the development of radiographic osteoarthritis among middle-aged and elderly persons: the Multicenter Osteoarthritis Study. Arthritis Rheumatol. 2009;60(3):831–839. 10.1002/art.24383 PMC2758243 19248082

[31] Fregly BJ Besier TF Lloyd DG Grand challenge competition to predict in vivo knee loads. J Orthop Res. 2012;30(4):503–513. 22161745 10.1002/jor.22023 PMC4067494

[32] Frobell RB Roos HP Roos EM Roemer FW Ranstam J Lohmander LS Treatment for acute anterior cruciate ligament tear: five year outcome of randomised trial. BMJ. 2013;346:F232. 23349407 10.1136/bmj.f232 PMC3553934

[33] Frost HM Skeletal structural adaptations to mechanical usage (SATMU): 1. Redefining Wolff’s law: the bone modeling problem. Anat Rec. 1990;226(4):403–413. 2184695 10.1002/ar.1092260402

[34] Gardiner BS Woodhouse FG Besier TF Predicting knee osteoarthritis. Ann Biomed Eng. 2016;44(1):222–233. 26206679 10.1007/s10439-015-1393-5 PMC4690844

[35] Gardinier ES Manal K Buchanan TS Snyder-Mackler L Minimum detectable change for knee joint contact force estimates using an EMG-driven model. Gait Posture. 2013;38(4):1051–1053. 23601782 10.1016/j.gaitpost.2013.03.014 PMC3795951

[36] Gerus P Sartori M Besier TF Subject-specific knee joint geometry improves predictions of medial tibiofemoral contact forces. J Biomech. 2013;46(16):2778–2786. 24074941 10.1016/j.jbiomech.2013.09.005 PMC3888900

[37] Gottlob CA Baker CL Jr Pellissier JM Colvin L Cost effectiveness of anterior cruciate ligament reconstruction in young adults. Clin Orthop Relat Res. 1999(367):272–282. 10546625

[38] Hagino T Ochiai S Senga S Meniscal tears associated with anterior cruciate ligament injury. Arch Orthop Trauma Surg. 2015;135(12):1701–1706. 26286641 10.1007/s00402-015-2309-4

[39] Hunter DJ Altman RD Cicuttini F OARSI clinical trials recommendations: knee imaging in clinical trials in osteoarthritis. Osteoarthritis Cartilage. 2015;23(5):698–715. 25952343 10.1016/j.joca.2015.03.012

[40] Jones G Ding C Glisson M Hynes K Ma D Cicuttini F Knee articular cartilage development in children: a longitudinal study of the effect of sex, growth, body composition, and physical activity. Pediatr Res. 2003;54(2):230–236. 12736391 10.1203/01.PDR.0000072781.93856.E6

[41] Kim YJ Bonassar LJ Grodzinsky AJ The role of cartilage streaming potential, fluid flow and pressure in the stimulation of chondrocyte biosynthesis during dynamic compression. J Biomech. 1995;28(9):1055–1066. 7559675 10.1016/0021-9290(94)00159-2

[42] Kiviranta I Jurvelin J Tammi M Saamanen AM Helminen HJ Weight bearing controls glycosaminoglycan concentration and articular cartilage thickness in the knee joints of young beagle dogs. Arthritis Rheum. 1987;30(7):801–809. 3619962 10.1002/art.1780300710

[43] Kiviranta I Tammi M Jurvelin J Saamanen AM Helminen HJ Moderate running exercise augments glycosaminoglycans and thickness of articular cartilage in the knee joint of young beagle dogs. J Orthop Res. 1988;6(2):188–195. 3278079 10.1002/jor.1100060205

[44] Konrath JM Vertullo CJ Kennedy BA Bush HS Barrett RS Lloyd DG Morphologic characteristics and strength of the hamstring muscles remain altered at 2 years after use of a hamstring tendon graft in anterior cruciate ligament reconstruction. Am J Sports Med. 2016;44(10):2589–2598. 27432052 10.1177/0363546516651441

[45] Koo S Alexander EJ Gold GE Giori NJ Andriacchi TP Morphology and thickness in tibial and femoral cartilage at the knee is influenced by the mechanics of walking (abstract). Paper presented at: ASME Summer Bioengineering Conference; June 2003; Miami, FL.

[46] Koo S Andriacchi TP A comparison of the influence of global functional loads vs. local contact anatomy on articular cartilage thickness at the knee. J Biomech. 2007;40(13):2961–2966. 17418219 10.1016/j.jbiomech.2007.02.005 PMC2358971

[47] Lebel B Hulet C Galaud B Burdin G Locker B Vielpeau C Arthroscopic reconstruction of the anterior cruciate ligament using bone-patellar tendon-bone autograft: a minimum 10-year follow-up. Am J Sports Med. 2008;36(7):1275–1282. 18354147 10.1177/0363546508314721

[48] Lee JH Kisiday J Grodzinsky AJ Tissue-engineered versus native cartilage: linkage between cellular mechano-transduction and biomechanical properties. Novartis Found Symp. 2003;249:52–64; discussion 64-59, 170-174, 239-141. 12708649

[49] Lee YS Jeong YM Sim JA Specific compartmental analysis of cartilage status in double-bundle ACL reconstruction patients: a comparative study using pre- and postoperative MR images. Knee Surg Sports Traumatol Arthrosc. 2013;21(3):702–707. 22592653 10.1007/s00167-012-2046-y

[50] Lloyd DG Besier TF An EMG-driven musculoskeletal model to estimate muscle forces and knee joint moments in vivo. J Biomech. 2003;36(6):765–776. 12742444 10.1016/s0021-9290(03)00010-1

[51] Lohmander LS Englund PM Dahl LL Roos EM The long-term consequence of anterior cruciate ligament and meniscus injuries: osteoarthritis. Am J Sports Med. 2007;35(10):1756–1769. 17761605 10.1177/0363546507307396

[52] Lohmander LS Ostenberg A Englund M Roos H High prevalence of knee osteoarthritis, pain, and functional limitations in female soccer players twelve years after anterior cruciate ligament injury. Arthritis Rheum. 2004;50(10):3145–3152. 15476248 10.1002/art.20589

[53] Magnussen RA Mansour AA Carey JL Spindler KP Meniscus status at anterior cruciate ligament reconstruction associated with radiographic signs of osteoarthritis at 5- to 10-year follow-up: a systematic review. J Knee Surg. 2009;22(4):347–357. 19902731 10.1055/s-0030-1247773 PMC3785109

[54] Miyasaka KC Daniel DM Stone ML Hirshman P The incidence of knee ligament injuries in the general population. Am J Knee Surg. 1991;4(1):3–8.

[55] Moksnes H Engebretsen L Risberg MA Prevalence and incidence of new meniscus and cartilage injuries after a nonoperative treatment algorithm for ACL tears in skeletally immature children: a prospective MRI study. Am J Sports Med. 2013;41(8):1771–1779. 23771955 10.1177/0363546513491092

[56] Mundermann A Payer N Felmet G Riehle H Comparison of volumetric bone mineral density in the operated and contralateral knee after anterior cruciate ligament and reconstruction: a 1-year follow-up study using peripheral quantitative computed tomography. J Orthop Res. 2015;33(12):1804–1810. 26123943 10.1002/jor.22962

[57] Neuman P Kostogiannis I Friden T Roos H Dahlberg LE Englund M Patellofemoral osteoarthritis 15 years after anterior cruciate ligament injury: a prospective cohort study. Osteoarthritis Cartilage. 2009;17(3):284–290. 18771938 10.1016/j.joca.2008.07.005

[58] Oiestad BE Engebretsen L Storheim K Risberg MA Knee osteoarthritis after anterior cruciate ligament injury: a systematic review. Am J Sports Med. 2009;37(7):1434–1443. 19567666 10.1177/0363546509338827

[59] Peterfy CG van Dijke CF Janzen DL Quantification of articular cartilage in the knee with pulsed saturation transfer subtraction and fat-suppressed MR imaging: optimization and validation. Radiology. 1994;192(2):485–491. 8029420 10.1148/radiology.192.2.8029420

[60] Pizzolato C Reggiani M Saxby DJ Ceseracciu E Modenese L Lloyd DG Biofeedback for gait retraining based on real-time estimation of tibiofemoral joint contact forces [published online April 18, 2017]. IEEE Trans Neural Syst Rehabil Eng. doi:10.1109/TNSRE.2017.2683488. 10.1109/TNSRE.2017.2683488 PMC5757380 28436878

[61] Rosset A Spadola L Ratib O OsiriX: an open-source software for navigating in multidimensional DICOM images. J Digit Imaging. 2004;17(3):205–216. 15534753 10.1007/s10278-004-1014-6 PMC3046608

[62] Ruano JS Sitler MR Driban JB Prevalence of radiographic knee osteoarthritis after anterior cruciate ligament reconstruction, with or without meniscectomy: an evidence-based practice paper. J Athl Train. 2017;52(6):606–609. 26930022 10.4085/1062-6050-51.2.14 PMC5488852

[63] Saxby DJ Bryant AL Modenese L Tibiofemoral contact forces in the anterior cruciate ligament-reconstructed knee. Med Sci Sports Exerc. 2016;48(11):2195–2206. 27337173 10.1249/MSS.0000000000001021

[64] Saxby DJ Modenese L Bryant AL Tibiofemoral contact forces during walking, running and sidestepping. Gait Posture. 2016;49:78–85. 27391249 10.1016/j.gaitpost.2016.06.014

[65] Scanlan SF Chaudhari AM Dyrby CO Andriacchi TP Differences in tibial rotation during walking in ACL reconstructed and healthy contralateral knees. J Biomech. 2010;43(9):1817–1822. 20181339 10.1016/j.jbiomech.2010.02.010 PMC2882513

[66] Shelbourne KD Nitz P Accelerated rehabilitation after anterior cruciate ligament reconstruction. Am J Sports Med. 1990;18(3):292–299. 2372081 10.1177/036354659001800313

[67] Shi DL Wang YB Ai ZS Effect of anterior cruciate ligament reconstruction on biomechanical features of knee in level walking: a meta-analysis. Chin Med J (Engl). 2010;123(21):3137–3142. 21162970

[68] Su F Hilton JF Nardo L Cartilage morphology and T1rho and T2 quantification in ACL-reconstructed knees: a 2-year follow-up. Osteoarthritis Cartilage. 2013;21(8):1058–1067. 23707754 10.1016/j.joca.2013.05.010 PMC3752987

[69] Van Rossom S Smith CR Zevenbergen L Knee cartilage thickness, T1rho and T2 relaxation time are related to articular cartilage loading in healthy adults. PLoS One. 2017;12(1):E0170002. 28076431 10.1371/journal.pone.0170002 PMC5226797

[70] Wang X Wang Y Bennell KL Cartilage morphology at 2-3 years following anterior cruciate ligament reconstruction with or without concomitant meniscal pathology. Knee Surg Sports Traumatol Arthrosc. 2017;25(2):426–436. 26506844 10.1007/s00167-015-3831-1

[71] Wang Y Wluka AE Berry PA Increase in vastus medialis cross-sectional area is associated with reduced pain, cartilage loss, and joint replacement risk in knee osteoarthritis. Arthritis Rheum. 2012;64(12):3917–3925. 23192791 10.1002/art.34681

[72] Wellsandt E Gardinier ES Manal K Axe MJ Buchanan TS Snyder-Mackler L Decreased knee joint loading associated with early knee osteoarthritis after anterior cruciate ligament injury. Am J Sports Med. 2016;44(1):143–151. 26493337 10.1177/0363546515608475 PMC4703470

[73] Williams GN Snyder-Mackler L Barrance PJ Axe MJ Buchanan TS Muscle and tendon morphology after reconstruction of the anterior cruciate ligament with autologous semitendinosus-gracilis graft. J Bone Joint Surg Am. 2004;86-A(9):1936–1946. 15342756 10.2106/00004623-200409000-00012

[74] Winby CR Lloyd DG Besier TF Kirk TB Muscle and external load contribution to knee joint contact loads during normal gait. J Biomech. 2009;42(14):2294–2300. 19647257 10.1016/j.jbiomech.2009.06.019

[75] Zabala ME Favre J Scanlan SF Donahue J Andriacchi TP Three-dimensional knee moments of ACL reconstructed and control subjects during gait, stair ascent, and stair descent. J Biomech. 2013;46(3):515–520. 23141637 10.1016/j.jbiomech.2012.10.010 PMC3552088

